# Interim 2017/18 influenza seasonal vaccine effectiveness: combined results from five European studies

**DOI:** 10.2807/1560-7917.ES.2018.23.9.18-00086

**Published:** 2018-03-01

**Authors:** Marc Rondy, Esther Kissling, Hanne-Dorthe Emborg, Alin Gherasim, Richard Pebody, Ramona Trebbien, Francisco Pozo, Amparo Larrauri, Jim McMenamin, Marta Valenciano, I-MOVE/I-MOVE+ group

**Affiliations:** 1 EpiConcept, Paris, France; 2 MR and EK contributed equally to the study and manuscript writing; 3 Department of Infectious Disease Epidemiology and Prevention, Statens Serum Institut, Copenhagen, Denmark; 4 National Epidemiology Centre, Institute of Health Carlos III, Madrid Spain; CIBER de Epidemiología y Salud Pública (CIBERESP), Institute of Health Carlos III, Madrid Spain; 5 Public Health England, London, United Kingdom; 6 Department of Virus and Microbiological Special diagnostics, National Influenza Center, Statens Serum Institut, Copenhagen, Denmark; 7 Inmaculada Casas National Centre for Microbiology, National Influenza Reference Laboratory, World Health Organization National Influenza Centre, Institute of Health Carlos III; 8 Health Protection Scotland, Glasgow, United Kingdom; 9 The members of the I-MOVE/I-MOVE+ group are listed at the end of the article

**Keywords:** influenza, influenza vaccine effectiveness, influenza vaccination, case control study, multicentre study, Europe

## Abstract

Between September 2017 and February 2018, influenza A(H1N1)pdm09, A(H3N2) and B viruses (mainly B/Yamagata, not included in 2017/18 trivalent vaccines) co-circulated in Europe. Interim results from five European studies indicate that, in all age groups, 2017/18 influenza vaccine effectiveness was 25 to 52% against any influenza, 55 to 68% against influenza A(H1N1)pdm09, −42 to 7% against influenza A(H3N2) and 36 to 54% against influenza B. 2017/18 influenza vaccine should be promoted where influenza still circulates.

Most countries in the European Union (EU) recommend and fund seasonal influenza vaccine for elderly people and individuals at increased risk of severe influenza [1]. The United Kingdom (UK) commenced the incremental introduction of a universal childhood influenza vaccination programme in 2013/14 using a quadrivalent live attenuated influenza vaccine (LAIV4) for healthy children and quadrivalent inactivated vaccine (QIV) for at-risk children for whom LAIV4 is contraindicated [2].

The trivalent influenza vaccines for the 2017/18 northern hemisphere influenza season include an A/Michigan/45/2015 (H1N1)pdm09-like virus, an A/Hong Kong/4801/2014 (H3N2)-like virus and a B/Brisbane/60/2008-like virus (B/Victoria lineage). The quadrivalent vaccines also contain a B/Phuket/3073/2013-like virus (B/Yamagata lineage) [3].

The early phase of the 2017/18 influenza season in Europe was characterised by the co-circulation of influenza A(H1N1)pdm09, influenza A(H3N2) and influenza B, with different patterns of dominant type or subtype observed between countries [4]. Up to February 2018, most influenza B viruses assigned to a lineage were B/Yamagata viruses, not included in the 2017/18 trivalent vaccine [3,4].

Here we present the interim 2017/18 season influenza vaccine effectiveness (VE) estimates from three single-country studies (UK, Denmark (DK) and Spain (ES)) and two multi-country studies (primary care (EU-PC) and hospital (EU-H) European Influenza - Monitoring Vaccine Effectiveness (I-MOVE/I-MOVE+) networks) (Figure 1).

**Figure 1:**
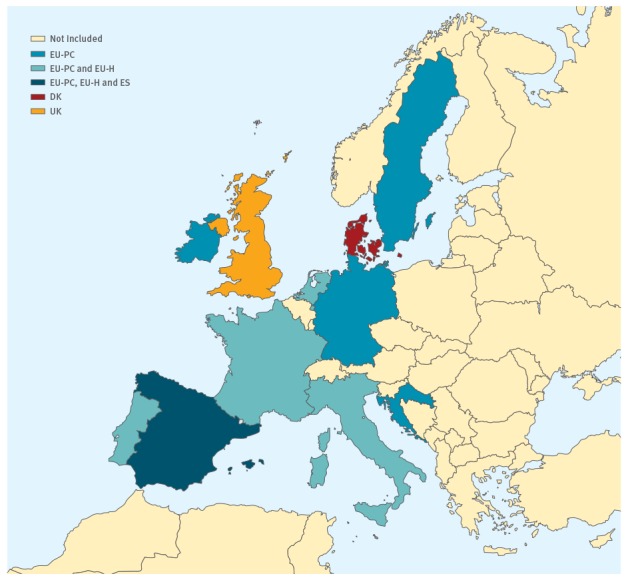
European Union countries contributing to the influenza vaccine effectiveness results presented, by study, 2017/18 (n = 21,220)

DK: Denmark study; ES: Spain study; EU-H: European hospital-based multi-country I-MOVE+ study; EU-PC: European primary care-based multi-country I-MOVE/I-MOVE+ study; UK: United Kingdom study.

## Study design and estimation of vaccine effectiveness

The methods of these five studies have been described in detail elsewhere [5-9]. Study sites included in EU-PC (Croatia, France, Germany, Ireland, Italy, the Netherlands, Portugal, Spain and Sweden) and EU-H (France, Italy, the Netherlands, Portugal and Spain) followed generic protocols for primary care-based or hospital-based studies. 

All five studies used a test-negative case control design (TND) [10]. In short, individuals presenting at participating healthcare settings with a pre-determined set of symptoms (including at least one systemic and one respiratory symptom) were swabbed. Samples were tested for influenza using RT-PCR. Individuals testing positive for influenza were classified as cases (by influenza (sub)type), those testing negative as controls.

The ES, UK and EU-PC studies included patients at primary care level (henceforth referred to as medically attended), the EU-H study included patients at hospital level (henceforth referred to as hospitalised), and the DK study included results from primary care and hospital level pooled together. Patients’ inclusion was foreseen to be systematic (or exhaustive) in the ES, EU-PC and EU-H studies and ad hoc in the DK and UK studies. In Spain, 268 of the 833 physicians included in the ES study were also included in the EU-PC study.

The study population included all age groups in all studies except for EU-H, which was confined to individuals 65 years and older.

In all studies, we defined patients as vaccinated with the 2017/18 influenza vaccine if they had been vaccinated at least 14 days (UK) or 15 days (all other studies) before symptom onset. Patients were excluded if they were vaccinated less than 14 (UK) or 15 days (all other studies) before symptom onset or if the date of vaccination was unknown.

In seven EU-PC countries (France, Germany, Ireland, the Netherlands, Portugal, Spain and Sweden), the UK, ES and DK, all or a random sample of positive influenza specimens were selected for genetic sequencing.

VE was computed by comparing the odds of vaccination between cases and controls (VE = (1 − OR) × 100). All studies used logistic regression to adjust VE for measured confounding variables, excluding patients with missing data for covariates in the model (complete case analysis) (Table 1). We computed VE overall and, where possible, by age group and target population (as defined locally in the various studies and study sites) against any influenza, influenza A(H3N2), influenza A(H1N1)pdm09, any influenza B and influenza B/Yamagata.

**Table 1:** Summary characteristics of the influenza vaccine effectiveness studies included, Europe, influenza season 2017/18 (n = 21,220)

	ES	UK	EU-PC	DK	EU-H
Study period	30 Oct 2017 to 21 Jan 2018	1 Oct 2017 to 14 Jan 2018	25 Sep 2017 to 26 Jan 2018	1 Dec 2017 to 5 Feb 2018	25 Oct 2017 to 4 Feb 2018
Setting	Primary care	Primary care	Primary care	Primary care and hospital	Hospital
Location	Spain	England, Scotland, Northern Ireland and Wales	Croatia, France, Germany, Ireland, Italy, the Netherlands, Portugal, Spain and Sweden	Denmark	France, Italy, the Netherlands, Portugal and Spain
Study design	TND	TND	TND	TND	TND
Data source	Sentinel physicians and laboratory	Sentinel physicians and laboratory	Sentinel physicians and laboratory^a^	Data linkage of Danish Microbiology Database, the Danish Vaccination Register and the Danish National Hospital Register	Hospital charts, vaccine registers, interviews with GPs/pharmacists, laboratory
Age group of study population	All	All	≥ 6 months	≥ 18 years	≥ 65 years
Case definition	ILI	ILI	ILI	ILI	SARI
Selection of patients	Systematic	At practitioner's discretion	Systematic	At practitioner's discretion	Exhaustive
Vaccine types used^b^	Mostly TIV (no individual data)	In children: 19% TIV, 77% LAIV4 nasal spray, 3% unknown In adults: 100% unknown	67% TIV, 17% unknown, 8% TIV adjuvanted, 4% QIV, 3% TIV intradermal, 1% LAIV4 nasal spray	Only TIV (no individual data)	57% TIV, 25% unknown, 8% TIV adjuvanted, 10% TIV intradermal
Variables of adjustment	Age (RCS), sex, presence of chronic conditions, onset date, region	Age group, sex, onset date, pilot area for child vaccination programme, surveillance scheme	Age (RCS), sex, presence of chronic conditions, onset date and study site	Age group, sex, presence of chronic conditions, onset date	Age (RCS), lung diseases, heart diseases, diabetes mellitus, obesity (BMI ≥ 30), renal diseases, cancer and hospitalisation in the past 12 months, onset date, study site

DK: Denmark study; ES: Spain study; EU-H: European hospital-based multi-country I-MOVE+ study; EU-PC: European primary care-based multi-country I-MOVE/I-MOVE+ study; GP: general practitioner; ILI: influenza-like illness; LAIV4: quadrivalent live attenuated influenza vaccines; LRI: lower respiratory infection; SARI: severe acute respiratory infection; TIV: trivalent inactivated vaccines; TND: test-negative design; RCS: restricted cubic spline; UK: United Kingdom study.

^a^ In Spain, 268 of the 833 physicians included in the ES study were also included in the EU-PC study.

^b^ Vaccines were egg-propagated, non-adjuvanted and administered intramuscularly unless otherwise specified.

If the number of events per parameter was lower than 10, we conducted a sensitivity analysis using penalised logistic regression to assess small sample bias [11].

## Results

**Figure 2:**
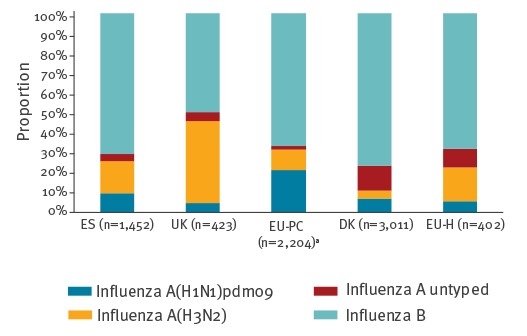
Proportion of influenza (sub)types by study, Europe, 2017/18 (n = 6,979)

DK: Denmark study; ES: Spain study; UK: United Kingdom study; EU-H: European hospital-based multi-country I-MOVE+ study; EU-PC: European primary care-based multi-country I-MOVE/I-MOVE+ study.

^a^ Includes two influenza A(H1N1)pdm09/B co-infections; 98 of influenza A(H1N1)pdm09, 122 of A(H3N2) and 454 of B cases were also in ES.

Between September 2017 and February 2018, the number of patients included in the VE analysis by study was 2,399 (1,452 cases) in the ES, 1,331 (421 cases) in the UK, 4,652 (2,103 cases) in the EU-PC, 11,907 (3,011 cases) in the DK and 931 (385 cases) in the EU-H study. Overall, more than two thirds of cases were positive for influenza B viruses in all studies except UK, where influenza A and B viruses were detected in similar proportions (51% (214/423) and 49% (209/423), respectively) (Figure 2). Where subtyped, influenza A viruses were mainly A(H3N2) in ES (62% (233/375) of subtyped influenza A specimens), UK (90% (174/194)) and EU-H (74% (68/92)), and mainly A(H1N1)pdm09 in DK (56% (145/257)) and EU-PC (67% (469/698)).

The graph includes individuals excluded from the VE analysis due to missing covariates.

### Any influenza

Among all ages, VE against any medically attended influenza ranged between 25% (95% confidence interval (CI): −10 to 48) in the UK study and 52% (95% CI: 29 to 67) in the ES study. In UK, VE of the LAIV4 was 53% (95% CI: −56 to 86) in children and VE of the inactivated vaccine was 18% (95% CI: −23 to 45) in adults (Table 2). Among the target groups for influenza vaccination, the VE was 36% (95% CI: 13 to 53) in EU-PC and 40% (95% CI: 1 to 63) in the ES study. In EU-H, VE against any hospitalised influenza in patients aged 65 years and older was 35% (95% CI: 13 to 51).

**Table 2:** Interim adjusted seasonal vaccine effectiveness against any laboratory-confirmed influenza, influenza A(H1N1)pdm09, A(H3N2) and B, by age group, target group for vaccination and by study, Europe, influenza season 2017/18

Influenza type/subtype and study site	Setting	Study population	Cases			Controls			Adjusted VE	95% CI
			All	Vacc	%	All	Vacc	%		
**Influenza A + B**										
ES	PC	All ages	1,452	98	7	947	75	8	52	29 to 67
		0–14 years	589	15	3	370	13	4	78	45 to 91
		15–64 years	762	44	6	511	40	8	54	23 to 73
		≥ 65 years	101	42	42	66	22	33	21	−93 to 68
		Target group^a^	278	72	26	180	50	28	40	1 to 63
UK	PC	All ages	421	93	22	910	190	21	25	−10 to 48
		2–17 years (LAIV4)	69	5	7	166	19	11	53	−56 to 86
		≥ 18 years (IIV)	347	87	25	630	163	26	18	−23 to 45
EU-PC	PC	All ages	2,103	210	10	2,549	272	11	38	20 to 52
		0–17 years	846	26	3	998	35	4	59	23 to 78
		18–64 years	1,021	74	7	1,288	109	8	34	5 to 54
		≥ 65 years	234	110	47	262	128	49	44	8 to 66
		Target group^a^	554	172	31	713	217	30	36	13 to 53
DK	PC and hospital	All ages	3,011	593	20	8,896	2299	26	34	25 to 41
		18–64 years	1,564	136	9	3,462	538	16	47	35 to 58
		≥ 65 years	934	447	48	3,089	1,688	55	23	10 to 34
EU-H	Hospital	≥ 65 years	385	200	52	546	332	61	35	13 to 51
**Influenza A(H1N1)pdm09**										
EU-PC	PC	All ages	444	14	3	1,999	195	10	68	42 to 83
		18–64 years	203	7	3	955	77	8	63	12 to 84
DK	PC and hospital	All ages	214	18	8	8,896	2299	26	55	23 to 74
		18–64 years	119	7	6	3,462	538	16	60	13 to 82
		≥ 65 years	26	11	42	3,089	1,688	55	37	−40 to 72
**Influenza A(H3N2)**										
ES	PC	All ages	233	22	9	947	75	8	7	−74 to 51
UK	PC	All ages	194	58	30	910	190	21	−27	−111 to 24
EU-PC	PC	All ages	220	35	16	1,505	147	10	−16	−96 to 31
		18–64 years	140	9	6	771	66	9	27	−62 to 67
DK	PC and hospital	All ages	122	53	43	8,896	2,299	26	-42	−116 to 7
		18–64 years	45	6	13	3,462	538	16	21	−95 to 68
		≥ 65 years	67	45	67	3,089	1,688	55	−65	−178 to 2
EU-H	Hospital	≥ 65 years	60	38	63	242	154	64	−1	−93 to 47
**Any influenza B**										
ES	PC	All ages	1,022	72	7	947	75	8	52	27 to 68
		0–17 years	440	10	2	370	13	4	83	54 to 94
		18–64 years	503	31	6	511	40	8	51	13 to 72
		≥ 65 years	79	31	39	66	22	33	15	−114 to 66
		Target group^a^	207	53	26	180	50	28	38	−5 to 63
UK	PC	All ages	209	33	16	910	190	21	54	24 to 72
EU-PC	PC	All ages	1,368	150	11	2,510	269	11	39	19 to 54
		0–17 years	562	21	4	980	35	4	58	15 to 79
		18–64 years	643	55	9	1,279	108	8	27	−9 to 51
		≥ 65 years	161	74	46	250	126	50	54	20 to 73
		Target group^a^	382	125	33	696	215	31	39	14 to 56
DK	PC and hospital	All ages	2,298	437	19	8,896	2,299	26	36	27 to 44
		18–64 years	1,220	111	9	3,462	538	16	44	30 to 56
		≥ 65 years	701	319	46	3,089	1,688	55	28	14 to 39
EU-H	Hospital	≥ 65 years	249	131	53	524	321	61	34	8 to 52
**Influenza B/Yamagata**										
ES	PC	All ages	84	4	5	993	81	8	77	14 to 94
EU-PC^b^	PC	All ages	395	34	9	2,065	206	10	49	19 to 67

CI: confidence interval; DK: Denmark study; ES: Spain study; EU-H: European hospital-based multi-country I-MOVE+ study; EU-PC: European primary care-based multi-country I-MOVE/I-MOVE+ study; PC: primary care; UK: United Kingdom study; Vacc: vaccinated; VE: vaccine effectiveness.

^a^ Groups targeted by seasonal influenza vaccination as defined locally in the studies and study sites.

^b^ Only includes study sites where lineage was available for all samples or where lineage was determined systematically.

Study sites included in the EU-H analysis: France, Italy, Navarra, the Netherlands, Portugal and Spain (except for influenza A(H3N2) analysis: Navarra and Spain only).

Study sites included in EU-PC analysis for all influenza and influenza B: Croatia, France, Germany, Ireland, Italy, the Netherlands, Portugal, Spain, Sweden. For analysis against A(H1N1)pdm09: France, Germany, Italy and Spain were included. For analysis against A(H3N2): France, Germany, Ireland, Spain and Sweden were included.

### Influenza A(H1N1)pdm09

All 76 influenza A(H1N1)pdm09 viruses sequenced belonged to clade 6B.1 (A/Michigan/45/2015) (Table 2). 

VE against influenza A(H1N1)pdm09 was 68% (95% CI: 42 to 83) and 55% (95% CI: 23 to 74) among all ages in the EU-PC and DK studies, respectively. Among 18–64 year-olds, it was 63% (95% CI: 12 to 84) and 60% (95% CI: 13 to 82) in the EU-PC and DK studies, respectively. Among those aged 65 years and older, it was 37% (95% CI: −40 to 72) in the DK study (Table 2).

### Influenza A(H3N2)

Of the 204 influenza A(H3N2) viruses sequenced, 63% (n = 129) belonged to genetic clade 3C.2a, 35% (n = 72) to 3C.2a1 and 1% (n = 3) to 3C.3a (Table 3). 

**Table 3:** Influenza viruses characterised by clade and study site, Europe, influenza season 2017/18 (n = 886)

	Clade	ES ^a^		UK		EU-PC^b^		DK^c^	
		n	%	n	%	n	%	n	%
Total influenza A(H1N1)		n = 142		n = 20		n = 469		n = 113	
Sequenced		28	100	10	100	25	100	23	100
**A/Michigan/45/2015**	**6B.1**	**28**	**100**	**10**	**100**	**25**	**100**	**23**	**100**
Total influenza A(H3N2)		n = 233		n = 174		n = 229		n = 144	
Sequenced		51	100	59	100	43	100	51	100
**A/HongKong/4801/2014**	**3C.2a**	**20**	**39**	**46**	**78**	**27**	**63**	**36**	**71**
**A/Singapore/INFIMH-16–0019/2016**	**3C.2a1**	**31**	**61**	**10**	**17**	**16**	**37**	**15**	**29**
**A/Switzerland/9715293/2013**	**3C.3a**	**0**	**0**	**3**	**5**	**0**	**0**	**0**	**0**
Total influenza B		n = 1,022		n = 209		n = 1,469		n = 625	
Sequenced		164	100	116	100	207	100	109	100
**B/Yamagata**		**136**	**83**	**116**	**100**	**198**	**96**	**109**	**100**
B/Phuket/3073/2013	3	136	100	0	0	198	100	109	100
**B/Victoria**		**28**	**17**	**0**	**0**	**9**	**4**	**0**	**0**
B/Norway/2409/2017	1A Δ(K162, N163)	20	71	0	0	5	56	0	0
B/Brisbane/60/2008	1A	8	29	0	0	4	44	0	0

^a^ 50 specimens from ES are also included in EU-PC data.

^b^ The specimens sequenced from Spain are originating from the entire National Influenza Surveillance System between weeks 44/2017 and 03/2018.

^c^ Sequence information is based on a sub-sample of influenza-positive samples received for surveillance at the National Influenza Center Denmark from week 40/2017 to 4/2018.

Among all ages, VE against influenza A(H3N2) ranged from −42% (95% CI: −116 to 7) in the DK and 7% (95% CI: −74 to 51) in the ES study. VE against hospitalisation for influenza A(H3N2) in patients aged 65 years and older was −1% (95% CI: −93 to 47) in EU-H (Table 2).

### Influenza B

Of the 596 influenza B viruses sequenced, 94% (n = 559) were B/Yamagata (all belonging to clade 3 influenza B/Phuket/3073/2013) and 6% (n = 37) were influenza B/Victoria (25 belonging to clade 1A Δ(K162, N163) and 12 belonging to clade 1A) (Table 3).

Among all ages, VE against influenza B ranged between 36% (95% CI: 27 to 44) in the DK and 54% (95% CI: 24 to 72) in the UK study. Age group-specific VE was lowest at 15% (95% CI: −114 to 66) among those aged 65 years and older and highest at 83% (95%CI: 54 to 94) in the 0–14-year age group in the ES study. VE was 34% (95% CI: 8 to 52) against hospitalised influenza B in EU-H and 28% (95% CI: 14 to 39) against medically attended and hospitalised influenza B in the DK study among those aged 65 years and older (Table 2). VE against influenza B/Yamagata was 77% (95% CI: 14 to 94) in the ES study and 49% (95% CI: 19 to 67) in EU-PC (Table 2).

### Sensitivity analyses

For all of the above analyses, sensitivity analyses for small sample size gave similar results (absolute difference ranging between 1% and 6%).

## Discussion

Interim results from five established influenza VE studies across Europe indicate that 2017/18 VE against all influenza ranged between 25 and 52% among all ages and between 36 and 40% in the targeted groups. VE was moderate to good against influenza A(H1N1)pdm09 among all ages (55 to 68%), poor against influenza A(H3N2) with all point estimates below 8% for all ages, and moderate against influenza B, with point estimates between 39 and 52% for all ages.

The good VE against medically attended influenza A(H1N1)pdm09 is consistent with historical data [12]. However, during the last influenza A(H1N1)pdm09 season in Europe (2015/16), the EU-PC VE of 33% against influenza A(H1N1)pdm09 in all age groups was lower than what we report here [13]. In the 2015/16 season, the influenza vaccine strain A/California/7/2009 (H1N1)pdm09 differed from the circulating strains which mainly belonged to the genetic subgroup 6B.1 (represented by A/Michigan/45/2015 (H1N1)pdm09). This 6B.1 strain was included in the 2017/18 vaccine and was identified in all A(H1N1)pdm09 samples sequenced in the study sites. The change in vaccine strain may have led to a better VE against A(H1N1)pdm09. More precise end-of-season estimates and results at the hospital level will help investigate this hypothesis.

The influenza A(H3N2) component included in the 2017/18 northern hemisphere vaccine was the same as in the 2016/17 northern hemisphere vaccine [14]. As anticipated based on EU-H 2016/17 results [15] and 2017 interim results from Australia [16], and as already reported in other published early estimates for the northern hemisphere [17], the VE against influenza A(H3N2) was low in participating study sites. In our studies, 63% of sequenced influenza A(H3N2) viruses belonged to the A/HongKong/4801/2014 vaccine strain genetic group (3C.2a) and 35% to the A/Singapore/INFIMH-16–0019/2016 clade (3C.2a1), which is the selected strain in the 2018 southern hemisphere and 2018/19 northern hemisphere influenza vaccines [18]. Small sample size limited VE estimation by clade and subclade, which will be a priority for end-of-season analyses. Our results further support the need for more effective interventions in older people, in whom the burden of influenza A(H3N2) is most notable and the VE, including against severe outcome, is the lowest [19]. Based upon recent cost-effectiveness work undertaken by Public Health England, the UK Joint Committee on Vaccination and Immunisation has advised that use of adjuvanted trivalent inactivated vaccines (TIV) in those aged 65 years and older would be both more effective and cost-effective than the non-adjuvanted trivalent or quadrivalent vaccines currently in use [20].

The interim VE against medically attended influenza B was moderate in the studies included here (36% to 54% among all ages), similar to recently published estimates from northern hemisphere countries [17,21,22]. It was moderate to good against medically attended influenza B in children (58% in EU PC and 83% in ES) and poorer at 34% against hospitalised outcome among adults 65 years and older. The vast majority (94%) of sequenced influenza B samples were of the B/Yamagata lineage, which was not included in the 2017/18 northern hemisphere TIV. VE was 77% and 49% against influenza B/Yamagata in the ES and EU-PC studies, respectively, suggesting important cross-lineage protection. 

The UK study was the only one to provide VE estimates for the quadrivalent vaccines. Vaccine effectiveness against any influenza among children was similar in the UK study (53%), where children receive LAIV4, and in the EU-PC study (59%), where most vaccinated children received TIV. However, it is difficult to compare these estimates against any influenza since the relative proportion of circulating (sub)types was different in the UK, where there was a higher proportion of circulating influenza A(H3N2) viruses, compared with most countries participating in the EU-PC study. In past seasons where circulating and vaccine lineages were different, contradictory results were observed [13,23,24]. Partial, but not full cross-protection between mismatched influenza B lineages has been suggested by two systematic reviews [25,26]. More precise end-of-season estimates by lineage, age group and vaccine type would be of added value to discuss cross-lineage protection and the added protection conferred by quadrivalent vaccines. Such information is relevant at a time when QIV is available in most European countries [27] and preferentially recommended in some [28].

End-of-season analyses are needed to verify the conclusions from these interim season results. A larger sample size should allow more precise estimates, especially in stratified analyses. Recent publications suggest a potentially strong (boosting or lowering) effect of previous vaccination on VE estimates [29,30] and end-of-season analyses should take this into account. Although TND is a well-recognised study design to measure VE, we cannot rule out bias from unmeasured confounding.

These early VE results from five studies were included in the Global Influenza VE (GIVE) report to help inform the World Health Organization vaccine strain selection committee meeting on 22 February 2018. For the 2018/19 northern hemisphere trivalent vaccine, this selection committee recommended to include the same influenza A(H1N1) component as in the 2017/18 northern hemisphere vaccine, an A/Singapore/INFIMH-16–0019/2016 (H3N2)-like virus and a B/Colorado/06/2017-like virus (B/Victoria/2/87 lineage) [18].

In the context of an influenza season with co-circulation of influenza A(H3N2), influenza A(H1N1)pdm09 and influenza B viruses mismatched with the trivalent vaccine, results from these five EU studies indicate a moderate VE against all influenza. Vaccination continues to be the most effective preventive measure against influenza and uptake of the 2017/18 trivalent or quadrivalent influenza vaccines should still be promoted in countries with ongoing virus circulation. In particular in settings with evidence of influenza A(H3N2) virus circulation, prophylactic use of antiviral drugs, administered according to country-specific guidelines, could help prevent severe outcomes [31]. Based on our results and in the absence of major antigenic drift, we may expect a good protection of the 2018/19 northern hemisphere seasonal influenza vaccine against influenza A(H1N1) and B viruses. Monitoring the effectiveness of the 2018 southern influenza vaccine against influenza A(H3N2) viruses will be important to prepare for the next influenza season in the northern hemisphere.

## Notes

**I-MOVE/I-MOVE+ group:** Croatia EU-PC study: Bernard Kaic, Croatian Institute of Public Health, Zagreb Sanja Kurecic Filipovic, Croatian Institute of Public Health, Zagreb Vesna Visekruna-Vucina, Croatian Institute of Public Health, Zagreb Iva Pem Novosel, Croatian Institute of Public Health, Zagreb Zvjezdana Lovric, Croatian Institute of Public Health, Zagreb Goranka Petrović, Croatian Institute of Public Health, Zagreb Denmark DK study: Tyra Grove Krause, Department of Infectious Disease Epidemiology and Prevention, Statens Serum Institut, Artillerivej 5, 2300 Copenhagen S, Denmark Thea Kølsen Fische, Department of Virus and Microbiological Special diagnostics, National Influenza Center, Statens Serum Institut, Artillerivej 5, 2300 Copenhagen S, Denmark France EU-PC and EU-H studies: Bruno Lina, Laboratoire de Virologie, CNR des virus des infections respiratoires, Institut des Agents Infectieux, Groupement Hospitalier Nord des HCL, Lyon, France; Laboratoire Virpath, CIRI Inserm U1111, CNRS 5308, ENS, UCBL, Faculté de Médecine LYON Est, Université de Lyon, Lyon. EU-PC study: Alessandra Falchi, EA7310, Laboratoire de Virologie, Université de Corse-Inserm, FR- 20250, Corte, France Ana-Maria Vilcu, Cécile Souty, Thierry Blanchon, Sorbonne Université, INSERM, Institut Pierre Louis d’Epidémiologie et de Santé Publique (IPLESP), F75012 Paris. Sylvie van der Werf, Unité de Génétique Moléculaire des Virus à ARN, UMR 3569 CNRS, Université Paris Diderot SPC, Institut Pasteur, Paris; CNR des virus des infections respiratoires, Institut Pasteur, Paris Vincent Enouf, Unité de Génétique Moléculaire des Virus à ARN, UMR 3569 CNRS, Université Paris Diderot SPC, Institut Pasteur, Paris ; CNR des virus des infections respiratoires, Institut Pasteur, Paris. Sylvie Behillil, Unité de Génétique Moléculaire des Virus à ARN, UMR 3569 CNRS, Université Paris Diderot SPC, Institut Pasteur, Paris; CNR des virus des infections respiratoires, Institut Pasteur, Paris. Martine Valette, Laboratoire de Virologie, CNR des virus des infections respiratoires, Institut des Agents Infectieux, Groupement Hospitalier Nord des HCL, Lyon. Sibylle Bernard-Stoecklin, Santé publique France, Paris. Daniel Lévy-Bruhl, Santé publique France, Paris. EU-H study: Odile Launay, Innovative clinical research network in vaccinology, I-REIVAC, CIC Cochin-Pasteur, Paris Pierre Loulergue, I-REIVAC, CIC Cochin-Pasteur, Paris Nezha Lenzi, I-REIVAC, Paris Zineb Lesieur, I-REIVAC, Paris Anne-Sophie L'Honneur, service de Virologie, Hôpital Cochin, Paris Florence Galtier: CIC 1411, hôpital St Eloi, CHU de Montpellier ; Inserm, F-CRIN, I-REIVAC Camille Agostini, CIC 1411, hôpital St Eloi, CHU de Montpellier Chris Serrand, CIC 1411, hôpital St Eloi, CHU de Montpellier Corinne Merle, Service des Maladies Infectieuses et Tropicales, Hôpital Gui de Chauliac, CHU de Montpellier Vincent Foulongne, Pathogenesis and Control of Chronic Infections, Univ. Montpellier, INSERM, EFS, CHU Montpellier, Montpellier, France Philippe Vanhems, I-REIVAC; Hôpital Edouard Herriot, Lyon Fabrice Lainé, I-REIVAC; CIC 1414, Hôpital Pontchaillou, Rennes Gisèle Lagathu, laboratoire de virologie, C.H.U de Rennes Fabrice Carrat, UPMC Univ Paris 06, IPLESP UMRS 1136, Public health department, Hôpital Saint-Antoine, Paris Germany EU-PC study: Silke Buda, Department for Infectious Disease Epidemiology, Robert Koch Institute, Berlin Ute Preuss, Department for Infectious Disease Epidemiology, Robert Koch Institute, Berlin Kerstin Prahm, Department for Infectious Disease Epidemiology, Robert Koch Institute, Berlin Brunhilde Schweiger, National Reference Center for Influenza, Robert Koch Institute, Berlin Marianne Wedde, National Reference Center for Influenza, Robert Koch Institute, Berlin Alla Heider, National Reference Center for Influenza, Robert Koch Institute, Berlin Maria Martin, National Reference Center for Influenza, Robert Koch Institute, Berlin Barbara Biere, National Reference Center for Influenza, Robert Koch Institute, Berlin Ralf Duerrwald, National Reference Center for Influenza, Robert Koch Institute, Berlin Ireland EU-PC study: Lisa Domegan, HSE-Health Protection Surveillance Centre, Dublin Laura Coughlan, HSE-Health Protection Surveillance Centre, Dublin Joan O’Donnell, HSE-Health Protection Surveillance Centre, Dublin Michael Joyce, Irish College of General Practitioners, Dublin Claire Collins, Irish College of General Practitioners, Dublin Linda Dunford, National Virus Reference Laboratory, University College Dublin Joanne Moran, National Virus Reference Laboratory, University College Dublin Grainne Tuite, National Virus Reference Laboratory, University College Dublin Margaret Duffy, National Virus Reference Laboratory, University College Dublin Jeff Connell, National Virus Reference Laboratory, University College Dublin Cillian de Gascun, National Virus Reference Laboratory, University College Dublin Italy Italian Influenza Surveillance System: Caterina Rizzo, Istituto Superiore di Sanità, Rome Antonino Bella, Istituto Superiore di Sanità, Rome Valeria Alfonsi, Istituto Superiore di Sanità, Rome Maria Rita Castrucci, National Influenza Center, Istituto Superiore di Sanità, Rome Simona Puzelli, National Influenza Center, Istituto Superiore di Sanità, Rome EU-PC study: Elisabetta Pagani, Regional Reference Laboratory for influenza, TRENTINO ALTO ADIGE, Bolzano Valeria Ghisetti, Regional Reference Laboratory for influenza, PIEMONTE Elena Pariani, Regional Reference Laboratory for influenza, LOMBARDIA, Milano Fausto Baldanti, Regional Reference Laboratory for influenza, LOMBARDIA, Pavia Giorgio Palù, Regional Reference Laboratory for influenza, VENETO Pierlanfranco D'Agaro, Regional Reference Laboratory for influenza, FRIULI VENEZIA GIULIA Filippo Ansaldi, Regional Reference Laboratory for influenza, LIGURIA Paola Affanni, Regional Reference Laboratory for influenza, EMILIA-ROMAGNA Gian Maria Rossolini, Regional Reference Laboratory for influenza, TOSCANA Barbara Camilloni, Regional Reference Laboratory for influenza, UMBRIA Patrizia Bagnarelli, Regional Reference Laboratory for influenza, MARCHE Maurizio Sanguinetti, Regional Reference Laboratory for influenza, LAZIO Luigi Atripaldi, Regional Reference Laboratory for influenza, CAMPANIA Maria Chironna, Regional Reference Laboratory for influenza, PUGLIA Caterina Serra, Regional Reference Laboratory for influenza, SARDEGNA Francesco Vitale, Regional Reference Laboratory for influenza, SICILIA EU-H study: Maria Chironna, Department of Biomedical Science and Human Oncology, Bari hospital, Bari Cinzia Germinario, Department of Biomedical Science and Human Oncology, Bari hospital, Bari Andrea Orsi, San Martino Polyclinic Hospital, University of Genoa, Genoa Filippo Ansaldi, San Martino Polyclinic Hospital, University of Genoa, Genoa Ilaria Manini, Department of Molecular and Developmental Medicine, University of Siena, Siena Emanuele Montomoli, Department of Molecular and Developmental Medicine, University of Siena, Siena Christian Napoli, Sant’Andrea Hospital, Sapienza University of Rome, Rome Giovanni Battista Orsi, Sant’Andrea Hospital, Sapienza University of Rome, Rome Navarra: EU-H study: Itziar Casado, Instituto de Salud Pública de Navarra, IdiSNA, CIBERESP, Pamplona Jesús Castilla, Instituto de Salud Pública de Navarra, IdiSNA, CIBERESP, Pamplona Leticia Fernandino, Instituto de Salud Pública de Navarra, IdiSNA, CIBERESP, Pamplona Iván Martínez-Baz, Instituto de Salud Pública de Navarra, IdiSNA, CIBERESP, Pamplona Guillermo Ezpeleta, Instituto de Salud Pública de Navarra, IdiSNA, CIBERESP, Pamplona Ana Navascués, Complejo Hospitalario de Navarra, IdiSNA, Pamplona Alejandra Pérez-García, Complejo Hospitalario de Navarra, IdiSNA, Pamplona Aitziber Aguinaga, Complejo Hospitalario de Navarra, IdiSNA, Pamplona Carmen Ezpeleta, Complejo Hospitalario de Navarra, IdiSNA, Pamplona The Netherlands EU-PC and EU-H studies: Adam Meijer, National Institute for Public Health and the Environment (RIVM), Bilthoven Sharon van den Brink, National Institute for Public Health and the Environment (RIVM), Bilthoven Wim van der Hoek, National Institute for Public Health and the Environment (RIVM), Bilthoven Gabriel Goderski, National Institute for Public Health and the Environment (RIVM), Bilthoven Lisa Wijsman, National Institute for Public Health and the Environment (RIVM), Bilthoven EU-PC study: Mariam Bagheri, National Institute for Public Health and the Environment (RIVM), Bilthoven Frederika Dijkstra, National Institute for Public Health and the Environment (RIVM), Bilthoven Marit de Lange, National Institute for Public Health and the Environment (RIVM), Bilthoven Ton Marzec, National Institute for Public Health and the Environment (RIVM), Bilthoven Pieter Overduin, National Institute for Public Health and the Environment (RIVM), Bilthoven Anne Teirlinck, National Institute for Public Health and the Environment (RIVM), Bilthoven Erny Wentink, Netherlands Institute for Health Services Research (NIVEL), Utrecht Gé Donker, Netherlands Institute for Health Services Research (NIVEL), Utrecht EU-H study: Sierk Marbus, National Institute for Public Health and the Environment (RIVM), Bilthoven Rianne van Gageldonk- Lafeber, National Institute for Public Health and the Environment (RIVM), Bilthoven Peter Schneeberger, Jeroen Bosch Hospital, 's Hertogenbosch Jan Jelrik van Oosterheert, University Medical Center Utrecht, Utrecht Valentijn Schweitzer, University Medical Center Utrecht, Utrecht Geert Groeneveld, Leiden University Medical Center, Leiden Portugal EU-PC and EU-H studies: Baltazar Nunes, Departamento de Epidemiologia, Instituto Nacional de Saúde Dr. Ricardo Jorge, Lisbon Ausenda Machado, Departamento de Epidemiologia, Instituto Nacional de Saúde Dr. Ricardo Jorge, Lisbon Ana Paula Rodrigues, Departamento de Epidemiologia, Instituto Nacional de Saúde Dr. Ricardo Jorge, Lisbon Verónica Gomez, Departamento de Epidemiologia, Instituto Nacional de Saúde Dr. Ricardo Jorge, Lisbon Irina Kislaya, Departamento de Epidemiologia, Instituto Nacional de Saúde Dr. Ricardo Jorge, Lisbon EU-PC study: Raquel Guiomar, Departamento de Doenças Infeciosas, Instituto Nacional de Saúde Dr. Ricardo Jorge, Lisbon Pedro Pechirra, Departamento de Doenças Infeciosas, Instituto Nacional de Saúde Dr. Ricardo Jorge, Lisbon Paula Cristóvão, Departamento de Doenças Infeciosas, Instituto Nacional de Saúde Dr. Ricardo Jorge, Lisbon Inês Costa, Departamento de Doenças Infeciosas, Instituto Nacional de Saúde Dr. Ricardo Jorge, Lisbon EU-H study: António Panarra, Centro Hospitalar de Lisboa Central, Lisbon Rita Côrte-Real, Centro Hospitalar de Lisboa Central, Lisbon José Poças, Centro Hospitalar de Setúbal, Setúbal Maria João Peres, Centro Hospitalar de Setúbal, Setúbal Spain The cycEVA work group Amparo Larrauri:, National Centre of Epidemiology, Institute of Health Carlos III, CIBERESP Alin Gherasim, National Centre of Epidemiology, Institute of Health Carlos III, CIBERESP Francisco Pozo, Inmaculada Casas National Centre for Microbiology, National Influenza Reference Laboratory, HO-National Influenza Centre, Institute of Health Carlos III Luis García Comas, Dirección General de Salud Pública, Comunidad de Madrid María Esther Insua Marisquerena, Dirección General de Salud Pública, Comunidad de Madrid Juan Carlos Galán, Laboratorio Hospital Ramón y Cajal Mª Dolores Folgueira, Laboratorio Hospital Doce de Octubre Fernando Gonzalez Carril, Departamento de Salud, Gobierno del País Vasco, Rosa Sancho Martínez, Departamento de Salud, Gobierno del País Vasco Gustavo Cilla Laboratorio Hospital Donostia, CIBERER Jesús Castilla, Navarra Institute for Health Research IdiSNA, Pamplona, CIBERESP Manuel García Cenoz, Navarra Institute for Health Research IdiSNA, Pamplona, CIBERESP Ana Navascués, Complejo Hospitalario de Navarra Carmen Quiñones Rubio, Dirección General de Salud Pública y Consumo de La Rioja Eva Martinez Ochoa, Dirección General de Salud Pública y Consumo de La Rioja Miriam Blasco, Laboratorio Hospital San Pedro de Logroño Jaume Gimenez Duran, Servicio de Epidemiología, Dirección General de Salud Pública, Mallorca Juana Maria Vanrell, Servicio de Epidemiología, Dirección General de Salud Pública, Mallorca Jordi Reina, Laboratorio del Hospital Son Espases, Mallorca Daniel Castrillejo, Servicio de Epidemiología. DGSC, Consejería de Bienestar Social y Sanidad, Ciudad Autónoma e Melilla The Spanish Influenza Sentinel Surveillance System: Amparo Larrauri, National Centre of Epidemiology, Institute of Health Carlos III, CIBERESP Alin Manuel Gherasim, National Centre of Epidemiology, Institute of Health Carlos III, CIBERESP Concha Delgado, National Centre of Epidemiology, Institute of Health Carlos III, CIBERESP Jesus Oliva National Centre of Epidemiology, Institute of Health Carlos III, CIBERESP Francisco Pozo, National Centre for Microbiology, National Influenza Reference Laboratory, WHO-National Influenza Centre, Institute of Health Carlos III. Inmaculada Casas, National Centre for Microbiology, National Influenza Reference Laboratory, WHO-National Influenza Centre, Institute of Health Carlos III. The Spanish IMOVE+ study: the hospitals component: Amparo Larrauri, National Centre of Epidemiology, Institute of Health Carlos III, CIBERESP Alin Manuel Gherasim, National Centre of Epidemiology, Institute of Health Carlos III, CIBERESP Francisco Pozo, National Centre for Microbiology, National Influenza Reference Laboratory, WHO-National Influenza Centre, Institute of Health Carlos III. Inmaculada Casas, National Centre for Microbiology, National Influenza Reference Laboratory, WHO-National Influenza Centre, Institute of Health Carlos III. Miriam García Dirección General de Salud Pública, Aragón; Miriam Latorre, Hospital Universitario Miguel Servet, Zaragoza Ana María Milagro Beamonte, Hospital Universitario Miguel Servet, Zaragoza Ana Martinez Sapiña, Hospital Universitario Miguel Servet, Zaragoza Madalen Oribe Amores, Subdirección de Salud Pública Gipuzkoa, País Vasco Amaia Aizpurúa, Laboratorio Hospital Donostia, CIBERER Gustavo Cilla, Laboratorio Hospital Donostia, CIBERER Milagrosa Montes, Laboratorio Hospital Donostia, CIBERER Sweden EU-PC study: Katherina Zakikhany, The Public Health Agency of Sweden, Stockholm Mia Brytting, The Public Health Agency of Sweden, Stockholm Åsa Wiman, The Public Health Agency of Sweden, Stockholm Annasara Carnahan, The Public Health Agency of Sweden, Stockholm United Kingdom UK study: Richard Pebody, Public Health England, London Fiona Warburton, Public Health England, London Abdelmajid Djennad, Public Health England, London Joanna Ellis, Public Health England, London Nick Andrews, Public Health England, London Diogo Marques, Health Protection Scotland, Glasgow Simon Cottrell, Public Health Wales, Cardiff Arlene Reynolds, Health Protection Scotland, Glasgow Rory Gunson, West of Scotland Specialist Virology Centre, Glasgow Monica Galiano, Public Health England, London Angie Lackenby, Public Health England, London Chris Robertson, University of Strathclyde, Glasgow Mark O’Doherty, Public Health Agency Northern Ireland, Belfast Mary Sinnathamby, Public Health England, London Ivelina Yonova, University of Surrey, Guildford, Royal College of General Practitioners, London Catherine Moore, Public Health Wales, Cardiff Muhammed Sartaj, Public Health Agency Northern Ireland, Belfast Simon de Lusignan, University of Surrey, Guildford, Royal College of General Practitioners, London Jim McMenamin, Health Protection Scotland, Glasgow Maria Zambon, Public Health England, London EpiConcept EU-PC and EU-H studies: Marta Valenciano, Alain Moren, EpiConcept, Paris EU-PC study: Esther Kissling, EpiConcept, Paris EU-H study: Marc Rondy, EpiConcept, Paris European Centre for Disease Prevention and Control (ECDC) Pasi Penttinen, European Centre for Disease Prevention and Control
